# A universal variational quantum eigensolver for non-Hermitian systems

**DOI:** 10.1038/s41598-023-49662-5

**Published:** 2023-12-15

**Authors:** Huanfeng Zhao, Peng Zhang, Tzu-Chieh Wei

**Affiliations:** 1 Department of Electrical and Computer Engineering, Stony Brook University, Stony Brook, 11794 USA; 2 C. N. Yang Institute for Theoretical Physics, Stony Brook University, Stony Brook, 11794 USA

**Keywords:** Engineering, Electrical and electronic engineering, Computer science, Quantum information

## Abstract

Many quantum algorithms are developed to evaluate eigenvalues for Hermitian matrices. However, few practical approach exists for the eigenanalysis of non-Hermintian ones, such as arising from modern power systems. The main difficulty lies in the fact that, as the eigenvector matrix of a general matrix can be non-unitary, solving a general eigenvalue problem is inherently incompatible with existing unitary-gate-based quantum methods. To fill this gap, this paper introduces a Variational Quantum Universal Eigensolver (VQUE), which is deployable on noisy intermediate scale quantum computers. Our new contributions include: (1) The first universal variational quantum algorithm capable of evaluating the eigenvalues of non-Hermitian matrices—Inspired by Schur’s triangularization theory, VQUE unitarizes the eigenvalue problem to a procedure of searching unitary transformation matrices via quantum devices; (2) A Quantum Process Snapshot technique is devised to make VQUE maintain the potential quantum advantage inherited from the original variational quantum eigensolver—With additional $O(log_{2}{N})$ quantum gates, this method efficiently identifies whether a unitary operator is triangular with respect to a given basis; (3) Successful deployment and validation of VQUE on a real noisy quantum computer, which demonstrates the algorithm’s feasibility. We also undertake a comprehensive parametric study to validate VQUE’s scalability, generality, and performance in realistic applications.

## Introduction

Eigenvalue calculation forms the backbone of a multitude of scientific and engineering disciplines, many of which necessitate the evaluation of eigenvalues for non-Hermitian matrices. Particularly, in power systems’ dynamic analysis, the eigenvalues of non-Hermitian matrices are critical for determining the system’s small signal stability and oscillation modes^1–3^. As the grid-connected renewable energy resources swiftly increase and reach a total capacity of over 3.4 TeraWatts globally^4^, most of which being wind farms and photovoltaic solar arrays, these intermittent, inertia-less renewable electricity injections, in addition to the weather and climate impacts as well as unforeseeable faults, are exposing unprecedented challenges on the stability and resilience of power grids worldwide. Consequently, there is an urgent need to evaluate eigenvalues for extremely large non-Hermitian matrices arising from power systems so as to enable fast assessment and assurance of power system stability and resilience.

The QR algorithm, underpinned by Schur’s decomposition, is the most commonly employed method for calculating eigenvalues^5^. However, it demands a computational complexity of $O(N^3)$, posing a significant challenge for analyzing large networks due to its scalability limitations. In pursuit of greater scalability, extensive efforts have been devoted to the development of iterative Krylov subspace eigenvalue algorithms, such as the Arnoldi algorithm^6^. By leveraging the sparsity property, these methods can efficiently compute a limited subset of eigenvalues, making them particularly the method of choice for large network analysis.

The fast development of quantum computation technologies carries a ray of light to forward this field further. For the eigenvalue evaluation problem of Hermitian matrice, the quantum phase estimation (QPE) method, for example, can find eigenvalues exponentially faster than the classical methods^7,8^; however, the probability distributions of eigenvalues it depends on the respective overlap of the initial states with eigenvectors. Recently, by generalizing the classical power method and employing techniques in the famous quantum linear system (known as HHL^9^), Nghiem and Wei^10^ introduce a quantum algorithm to estimate the largest eigenvalues of a Hermitian matrix, which also inherits the advantage of the HHL algorithm. Significant efforts have been expended in the last decade to extend the QPE method to the non-Hermitian matrix^11–13^. As shown in a recent research^14^, QPE is extended to evaluate the eigenvalues of a non-Hermitian but diagonalizable matrix, which either only has real eigenvalues or the corresponding input state could be prepared naturally. Furthermore, these methods all require an excessive long qubit decoherence time, which is unachievable on today’s noisy intermediate-scale quantum (NISQ) devices. The aforementioned problems are partially overcome by the variational quantum eigensolver (VQE) method proposed by Peruzzo et al.^15^. By enabling the quantum computer to work in concert with classical computer resources (e.g. optimizer), the long quantum process of finding eigenvalues can be broken up into a combination of multiple parameterized quantum programs (usually called ansatz). These ansatzes are shallow quantum circuits that only require a manageable coherent time. A classical optimizer assembles them globally to accomplish the eigenvalue evaluation. Such hybrid quantum-classical strategies are known as variational quantum algorithm (VQA) and have emerged as the leading technique to obtain a quantum advantage in the current and near future of NISQ era^16^. Such variational approaches work particularly well for obtaining the largest or smallest eigenvalues of a Hermitian matrix. Tremendous efforts are still being made to improve the VQE algorithms and ascertain their advantage. For instance, a large class of quantum algorithms are developed to enable VQE to search for excited states^17–21^. By exploiting the relation of diagonalization and majorization, Cerezo et al.^22^ introduces a more amenable method for estimating the largest eigenvalues. To improve the convergence and evaluation accuracy, Harwood et al.^23^ introduces a hybrid homotopy continuation algorithm. A more detailed VQE method evolution history can be found in the review papers by Cerezo et al. and Tilly et al.^16,24^. The eigenvalue algorithm is also extended to find the singular value of an arbitrary matrix^25,26^, as the singular value decomposition is realized by two unitary transformation matrices. Very recently, a variational method has been proposed^27^ to evaluate a specific eigenvalue of a non-Hermitian system (This paper appeared around the same time as our manuscript was submitted. We acknowledge a reviewer for bringing it to our attention.). This method requires simultaneous optimization of both the eigenvalue and the ansatz to yield an eigenvalue estimation near a pre-specified value. However, to estimate each unique eigenvalue, the entire optimization must be performed anew, rendering the process inefficient for determining multiple eigenvalues of a large matrix.

However, the gap remains between the developed quantum eigenvalue algorithms and real-world power system (and most other engineering systems) problems as all power systems, no matter direct current (DC), alternating current (AC) or hybrid, have non-Hermitian systems matrices. The primary challenge is that the eigenvectors of a non-Hermitian matrix can be non-unitary, leading to instances where the use of unitary gate-based quantum methods fails to identify the eigenvector matrix required for matrix diagonalization. Further, power system small signal analysis requires identifying all the complex eigenvalues to determine the oscillation modes of the power network. Therefore, few deployable quantum algorithm has been established thus far to perform eigenanalysis for an arbitrary matrix.

It is also worth noting that, by using the method presented in the HHL paper^9^, a non-Hermitian matrix can be converted to a Hermitian matrix for eigenvalue evaluation. However, the eigenvalues of this Hermitian matrix are related to the non-Hermitian matrix’s singular values. Since generally no relationship exists between eigenvalues and singular values, the eigenvalues of the non-Hermitian matrix are still unknown and cannot be obtained by methods designed for the latter.

It is also worth noting that a non-Hermitian matrix can be converted to a Hermitian matrix for enabling quantum computation, as introduced in the classic HHL work^9^. However, the eigenvalues of this resultant Hermitian matrix are related to the non-Hermitian matrix’s singular values. Since there is generally no relationship between eigenvalues and singular values, the eigenvalues of the non-Hermitian matrix are still unknown and cannot be obtained by methods designed for the latter.

In addition, classical VQE methods only give estimated eigenvalues, while no accuracy information is directly available. However, it is desirable to quantify the estimation errors for practical applications.

To address the aforementioned challenges, we devise a novel variational quantum universal eigensolver (VQUE) which empowers eigenanalysis of a generic matrix. Specifically, our contributions are threefold:

- By reexamining Schur’s theory, we unitarize the eigenvalue problem into a process of searching a unitary transformation matrix. This forms a cornerstone of a practical variational quantum algorithm capable of finding eigenvalues for non-Hermitian systems.
- We devise a quantum process snapshot method to determine if an N-dimensional matrix is triangular. Compared with the brute-force approach, it reduces the computational complexity from $O(N^2)$ to $O(log_{2}{N})$ gates. Thus the quantum advantage of VQUE is maintained. We also propose a novel approach to implement the non-unitary operator directly within the quantum process snapshot, thereby enabling VQUE to examine any arbitrary matrix.
- We define our cost function as the distance between the transformed matrix and a triangular matrix. This can be conveniently determined using the newly proposed quantum process snapshot technique. In contrast to the traditional VQE method, this cost function also doubles as a gauge for accuracy. Therefore, it enables the use of a shallower tentative ansatz in a trial-and-error process, which substantially enhances VQUE’s performance in real-world applications.

VQUE’s scalability, universality, and efficacy are validated on noise-free simulators and NISQ devices.

## Preliminaries

### Variational quantum eigensolver

To establish a link between the Variational Quantum Eigensolver (VQE) algorithm and the mathematical process of identifying the smallest eigenvalue of a Hermitian matrix, this subsection succinctly revisits the VQE method. We will employ matrix and vector notation for this recapitulation, as demonstrated below.

For a Hermitian matrix ${\varvec{H}}$ (e.g., hamiltonian matrix), its Rayleigh quotient is defined as :

1 $$\begin{aligned} \begin{aligned} R({{\varvec{H}}},{\vec {x}})={\vec {x}}^\dagger {\varvec{H}}{\vec {x}}/{\vec {x}}^\dagger {\vec {x}}, \end{aligned} \end{aligned}$$

where the superscript $\dagger$ denotes complex conjugate and, without loss of generality, and when ${\vec {x}}$ is taken as a vector with unit magnitude then the denominator can be omitted.

It can be shown that the Rayleigh quotient reaches its minimum value $R({\varvec{H}},{\vec {x}})_{\min }=\lambda _{\min }$, when ${\vec {x}}$ is the eigenvector corresponding to the eigenvalue $\lambda _{\min }$^5,28^.

Based on the mathematical theory above, the VQE method decomposes the Hermitian matrix as a linear combination of tensor products of Pauli operators^15,29^:

2 $$\begin{aligned} \begin{aligned} {\varvec{H}} = \sum \nolimits _{s=1}^{4^n} c_s {\varvec{\sigma _s}} = \sum \nolimits _{s=1}^{4^n} \frac{1}{2^n}\text {Tr}({\varvec{\sigma _s}} {\varvec{H}}) \left( \bigotimes \nolimits _{k=1}^{n} {\varvec{\sigma _{s,k}}}\right) , \end{aligned} \end{aligned}$$

where ${\varvec{\sigma _s}}$ denotes the *s*-th unitary matrix which is formed by $\bigotimes \nolimits _{k=1}^{n} {\varvec{\sigma _{s,k}}}$ and $c_s = \frac{1}{2^n}\text {Tr}( {\varvec{\sigma _sH}})$ is the *s*-th coefficient correspondingly; ${\varvec{\sigma _{s,k}}} \in \{{\varvec{\sigma _I=I,\sigma _x,\sigma _y,\sigma _z\}}}$ is the single-qubit Pauli operator in *s*-th unitary matrix for the *k*-th qubit.

Therefore, by applying a parameterized circuit ${\varvec{U}}(\varvec{\theta })$ to a fixed initial state such as $|0\cdots 0\rangle$, i.e., with the output vector ${\vec {x}}={\varvec{U}}(\varvec{\theta }){\vec {x}}_{0}$, the algorithm obtains an estimate ${\vec {x}}^\dagger {\varvec{\sigma _s}}{\vec {x}}={\vec {x}}_{0}^\dagger {\varvec{U}}(\varvec{\theta })^\dagger {\varvec{\sigma _s}}{\varvec{U}}(\varvec{\theta }){\vec {x}}_{0}$. The estimate is iteratively optimized by a classical controller, which changes the parameter $\varvec{\theta }$ to minimize the expectation value ${\vec {x}}^\dagger {\varvec{H}}{\vec {x}}$ (or $\langle x|H|x\rangle$ in the standard Dirac notation) obtained by measurements, which equals to:

3 $$\begin{aligned} \begin{aligned} {\vec {x}}^\dagger {\varvec{H}}{\vec {x}} = \sum \nolimits _{s=1}^{4^n} c_s (\vec {x_0}^\dagger {\varvec{U}}(\varvec{\theta })^\dagger {\varvec{\sigma _s}}{\varvec{U}}(\varvec{\theta })\vec {x_0}). \end{aligned} \end{aligned}$$

The quantum advantage is then obtained from evaluating each $\vec {x_0}^\dagger {\varvec{U}}(\varvec{\theta })^\dagger {\varvec{\sigma _s}}{\varvec{U}}(\varvec{\theta })\vec {x_0}$^15^ on a quantum computer. In a classical computer, calculating such matrix multiplication with *N* dimension takes $O(N^2)$ computation costs. As a comparison, a quantum computer has the capability to evaluate such terms (also known as evaluating the expectation value) by joint local measurement of each qubit, which is exponentially faster than the classical computer^8,30^.

However, all steps in the above procedure are based on the pre-assumption that the matrix ${\varvec{H}}$ is Hermitian, whose eigenvector matrix is unitary.

## Results

### Mathematical foundation

The critical aspect of enabling variational quantum algorithms to calculate the eigenvalues of a general matrix involves identifying a unitary transformation matrix that can distinctly expose the eigenvalues in a similar manner to the eigenvector matrix. In our devised variational quantum universal eigensolver (VQUE), we utilize the mathematical foundation of Schur’s decomposition theory as summarized below to ensure the existence of the solution:

An arbitrary square matrix ${\varvec{A}}$ can be transformed to a triangular matrix ${\varvec{T}}$ as:

4 $$\begin{aligned} \begin{aligned} {\varvec{A}}={\varvec{Q}}^\dagger {\varvec{T}}{\varvec{Q}}, \end{aligned} \end{aligned}$$

where ${\varvec{Q}}$ is a unitary matrix. As (4) belongs to the class of similarity transformation, matrix ${\varvec{T}}$ has the same spectrum as matrix ${\varvec{A}}$. On the other hand, since ${\varvec{T}}$ is triangular, its eigenvalues are explicitly listed as its diagonal entries^5^. This forms the mathematical basis for the use of variational quantum algorithms, which iteratively triangularize non-Hermitian matrices with unitary operators. Once a matrix has been triangularized, its eigenvalues can be readily determined by measuring the entries along the diagonal of the triangularized matrix.

### Quantum process snapshot

Given the above mathematical basis, the next question to address is when one can terminate the iterative process. We develop a quantum process snapshot technique to effectively measure the proximity of an N-dimensional matrix to a triangular matrix, and it only requires a computational cost of $O(\log _{2}N)$ gates. To ascertain if an *N*-dimensional matrix is an upper triangular one, we need to inspect its $N(N-1)/2$ entries below the diagonal. On a classical computer, this is a simple task, achieved by verifying if these entries are zero. However, in quantum computation, the matrix (or operator) elements are not directly observable without measurement. Therefore, a thorough check would involve evaluating $N(N-1)/2$ entries, necessitating at least $O(N^2)$ operations. Such an approach would eliminate any quantum advantage we might derive from quantum computing.

Inspired by the quantum process tomography technique^31^, we develop the quantum process snapshot. It captures all $N(N-1)/2$ characteristics by one single quantum circuit and thus is denoted as a “snapshot” of the quantum process.

For an *N*-dimensional matrix ${\varvec{M}}$ to be an upper triangular matrix, its entries below the diagonal should be zeroes, i.e., $M_{ij}=0,\, \text{ for }\,\,\, j=1,2,\ldots ,N-1\, \text{ and }\,\,\, i=j+1,j+2,\ldots ,N$. In quantum computation, it can be represented as:

5 $$\begin{aligned} |\langle e_i|{\varvec{M}}|e_j\rangle |^2=0, \end{aligned}$$

for $j=1,2,...N-1\,$ and $\, i=j+1,j+2...N$, where $|e_i\rangle$ is the $i^{th}$ computational basis.

**Figure 1:**
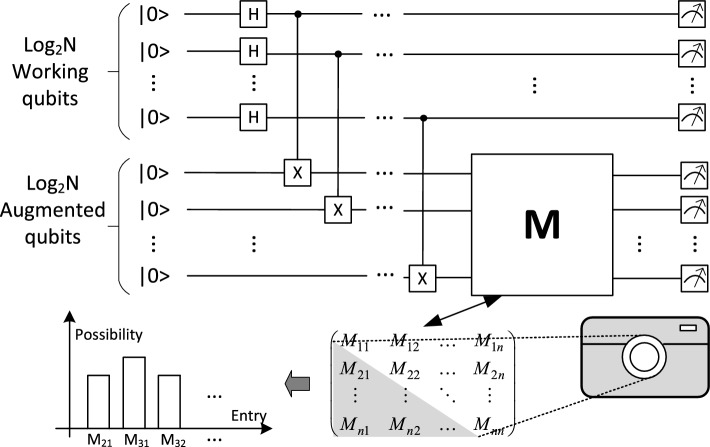
Quantum process snapshot.

This implies the possibility of measuring $|e_i\rangle$ from the quantum state ${\varvec{M}}|e_j\rangle$ should be zero, $\text{ for }\, j=1,2,\ldots ,N-1\, \text{ and }\,\, i=j+1,j+2,\ldots ,N$. Naively, to go through all quantum states $|e_j\rangle$, the procedure above needs to repeat *N*-1 times. By leveraging quantum parallelism and quantum interference, quantum states ${\varvec{M}}|e_1\rangle ,{\varvec{M}}|e_2\rangle ,\ldots ,{\varvec{M}}|e_N\rangle$ can be simultaneously encoded to one quantum state as shown in Fig. 1.

As this single circuit could capture all characteristics for determining its triangular property, we call it quantum process snapshot (QPS). Performing the ${\varvec{M}}$ operator to the augmented qubits gives the quantum states (6):

6 $$\begin{aligned} \frac{1}{\sqrt{N}}\sum _{i=1}^{N}{\varvec{I}}\otimes {\varvec{M}}|e_{i\_i}\rangle , \end{aligned}$$

where $|e_{i\_j}\rangle =|i\rangle \otimes |j\rangle$, which is the $(iN+j)^{th}$ computational basis.

This quantum state corresponds to the vector:

7 $$\begin{aligned} \begin{pmatrix} {\varvec{M}}\vec {e_1} \\ {\varvec{M}}\vec {e_2} \\ ...\\ {\varvec{M}}\vec {e_{N}} \end{pmatrix}, \end{aligned}$$

All matrix entry evaluations needed in (5) are then directly accessible from the measurement. For instance, the entry $M_{21}$ corresponding to the quantum measurement $|\langle e_2|{\varvec{M}}|e_1\rangle |^2$ can be evaluated by checking the possibility corresponding to the vector in (8). This vector corresponds to the measurement result of quantum state $|e_{0\_2}\rangle$ as defined in (6):

8 $$\begin{aligned} \begin{pmatrix} {\vec {e}}_{2} \\ {\vec {0}} \\ ...\\ {\vec {0}} \end{pmatrix}, \end{aligned}$$

Therefore, the QPS method could efficiently determine whether the implemented operator ${\varvec{M}}$ is triangular or not. However, in a quantum circuit, only the unitary operator is directly implementable, which only covers a small class of matrices (unitary matrix). This issue will be resolved in the next subsection, where we present a novel way to implement the non-unitary operator directly into the QPS process.

### Non-unitary gate operation

A novel method is devised for executing non-unitary gate operations within a unitary gate-based quantum machine. This innovative approach empowers variational quantum algorithms to compute the eigenvalues of any given matrix. In order to incorporate an arbitrary non-unitary operator ${\varvec{M}}$ into a quantum circuit, we start by decomposing it into a linear combination of unitaries, i.e., ${\varvec{M}}=\sum _{i}^{}c_i{\varvec{A}}_i$, with real number coefficients $c_i$ and a set of unitary operators ${\varvec{A}}_i$. We note that such decomposition into sum of unitaries was originally proposed by Childs and Wiebe in Hamiltonian simulations^32^. In this paper, (2) is assumed for such decomposition, but we note that other efficient decomposition of a matrix to unitaries do exist^33^. However, exploring other ways of decomposition is beyond the scope of this paper.

In the original VQE method, to efficiently evaluate the expectation value of a Hamiltonian by a quantum device, the matrix ${\varvec{H}}$ is decomposed as a linear combination of the simple Pauli operators (which are also unitary). The quantum expectation evaluation is then applied to each Pauli operator term ${\varvec{U}}(\varvec{\theta })^*{\varvec{\sigma _s}}{\varvec{U}}(\varvec{\theta })$. However, such implementation in QPS is not enough to determine a triangular matrix. This is because an independent implementation of unitary operators could only provide the value of each $(|\langle e_l|c_i{\varvec{A}}_i|e_m\rangle |^2)$. However, the summation of these magnitude squared values is mathematically different from the magnitude squared value of the summation $\sum _{i}^{}\langle e_l|c_i{\varvec{A}}_i|e_m\rangle$, which is the requirement as shown in (5),i.e.,

9 $$\begin{aligned} \begin{aligned} |\langle e_l|{\varvec{T}}|e_m\rangle |^2=(|\langle e_l|\sum _{i}^{}c_i{\varvec{A}}_i|e_m\rangle |^2), \end{aligned} \end{aligned}$$

10 $$\begin{aligned} \begin{aligned} (|\langle e_l|\sum _{i}^{}c_i{\varvec{A}}_i|e_m\rangle |^2)\not =\sum _{i}^{}|c_i|^2(|\langle e_l|{\varvec{A}}_i|e_m\rangle |^2). \end{aligned} \end{aligned}$$

As a comparison, in this subsection, we will adapt and modify the method introduced by Berry and Childs^34^, which implements the non-unitary gate operations directly.

Similar ideas to implement a non-unitary operator to a quantum state $|\psi \rangle$ are summarized by Childs et al.^35^. As an example, consider a non-unitary operator (matrix) ${\varvec{M}}=({\varvec{U_1}}+{\varvec{U_2}})$. To implement ${\varvec{M}}$ on a state $|\psi \rangle$, by adding one ancilla qubit, we start with the quantum state $|0\rangle \otimes |\psi \rangle$. Applying the Hadamard gate to the first qubit gives $\frac{1}{\sqrt{2}}(|0\rangle +|1\rangle )\otimes |\psi \rangle$. We then perform the controlled unitary gate $|0\rangle \langle 0|\otimes {\varvec{U_1}}+|1\rangle \langle 1|\otimes {\varvec{U_2}}$, giving the state $\frac{1}{\sqrt{2}}(|0\rangle \otimes {\varvec{U_1}}|\psi \rangle +|1\rangle \otimes {\varvec{U_2}}|\psi \rangle )$. In the end, by applying Hadamard gate to the ancilla qubit, we end up with the quantum state $\frac{1}{2} [ (|0\rangle \otimes ({\varvec{U_1}}+{\varvec{U_2}})|\psi \rangle +$ $|1\rangle \otimes ({\varvec{U_1}}-{\varvec{U_2}})|\psi \rangle ]$. If we measure the first qubit and obtain the $|0\rangle$ outcome, then we successfully prepare the qubit in the state $({\varvec{U_1}}+{\varvec{U_2}})|\psi \rangle$. Therefore, although all gate operations used are unitary, the non-unitary operation can still be implemented to a quantum state with an ancilla qubit. The non-unitary operation is essentially coded under a sub-state relating to ancilla qubit.

Berry and Childs^34^ and Childs and Wiebe^32^ use this idea to simulate hamiltonian dynamics of quantum state $\psi$, where the Hamiltonian is decomposed in the form of ${\varvec{M}}=\sum _{i=0}^{2^n-1}c_i{\varvec{A}}_i$, where $c_i$ is a positive constant number and ${\varvec{A}}_i$ is a unitary operator. Firstly, a unitary operator ${\varvec{B}}$ applied to the *n* ancilla qubits is defined as^34^:

11 $$\begin{aligned} \begin{aligned} {\varvec{B}}|0\rangle =\frac{1}{\sqrt{s}}\sum _{i=0}^{2^n-1}\sqrt{c_i}|i\rangle , \end{aligned} \end{aligned}$$

where $s=\sum _{i=0}^{2^n-1}c_i$. It is also important to realize that although two possible solutions exist for each $\sqrt{c_i}$, either of them could be selected to prepare the state. They will result in the same final output which is related to $(\sqrt{c_i})^*\cdot \sqrt{c_i}$, where the superscript * denotes complex conjugation.

Each unitary operator ${\varvec{A_i}}$ in ${\varvec{M}}=\sum _{i=0}^{2^n-1}c_i{\varvec{A}}_i$ is then implemented by the controlled operator *select*(*A*) defined as:

12 $$\begin{aligned} \begin{aligned} select(A)|i\rangle \otimes |\psi \rangle =|i\rangle \otimes {\varvec{A_j}}|\psi \rangle . \end{aligned} \end{aligned}$$

Therefore, applying the unitary operator ${\varvec{W}}=({\varvec{B}}^\dagger \otimes {\varvec{I}})(select(A))({\varvec{B}}\otimes {\varvec{I}})$ to the state $|0\rangle \otimes |\psi \rangle$ gives^34^:

13 $$\begin{aligned} \begin{aligned} {\varvec{W}}(|0\rangle \otimes |\psi \rangle )=\frac{1}{s}|0\rangle \otimes {\varvec{M'}}|\psi \rangle +\sqrt{1-\frac{1}{s^2}}|\phi \rangle , \end{aligned} \end{aligned}$$

where ${\varvec{M'}}=\sum _{i=0}^{2^n-1}|c_i|{\varvec{A}}_i$ and the ancillary state in state $|\phi \rangle$ is supported in the subspace orthogonal to $|0\rangle$. When the ancilla qubits are measured in state $|0\rangle$, the working qubits are in state ${\varvec{M'}}|\psi \rangle$.

From (13), it can be seen that the implemented operator ${\varvec{M'}}$ only equals the attempted non-unitary operator ${\varvec{M}}=\sum _{i=0}^{2^n-1}c_i{\varvec{A}}_i$, when all coefficient $c_i$ is real and non-negative. However, in general, the unitary decomposition in (2) will have negative or even complex $c_i$. To enable negative weights in the decomposition as shown (2), the controlled operator ${\varvec{W}}$ needs to be modified as below:

14 $$\begin{aligned} \begin{aligned} {\varvec{W_m}}=({\varvec{B_c}}^\dagger \otimes {\varvec{I}})(select(A))({\varvec{B}}\otimes {\varvec{I}}), \end{aligned} \end{aligned}$$

where the operator ${\varvec{B_c}}$ is defined as:

15 $$\begin{aligned} \begin{aligned} {\varvec{B_c}}|0\rangle =\frac{1}{\sqrt{s}}\sum _{i=0}^{2^n-1}(\sqrt{c_i})^*|i\rangle , \end{aligned} \end{aligned}$$

where the superscript * denotes complex conjugate.

Apply the modified operator to the quantum state $|0\rangle \otimes |\psi \rangle$ gives:

16 $$\begin{aligned} \begin{aligned} {\varvec{W_m}}(|0\rangle \otimes |\psi \rangle )=\frac{1}{s}|0\rangle \otimes {\varvec{M}}|\psi \rangle +\sqrt{1-\frac{1}{s^2}}|\phi \rangle , \end{aligned} \end{aligned}$$

where $s=\sum _{j=0}^{2^n-1}|c_i|$ and the ancillary state in state $|\phi \rangle$ is supported in the subspace orthogonal to $|0\rangle$. In such implementation, instead of $(\sqrt{c_i})^*\cdot \sqrt{c_i}=|c_i|$, the coefficient corresponding to the unitary matrix ${\varvec{A}}_i$ will become $(\sqrt{c_i})\cdot \sqrt{c_i}=c_i$. This makes the implemented operator always the same as ${\varvec{M}}=\sum _{i=0}^{2^n-1}c_i{\varvec{A}}_i$. The mathematical proof of (16) can be seen in section ‘Proof of non-unitary gate operation implementation’ of ‘Methods’.

### Variational quantum universal eigensolver

**Figure 2:**
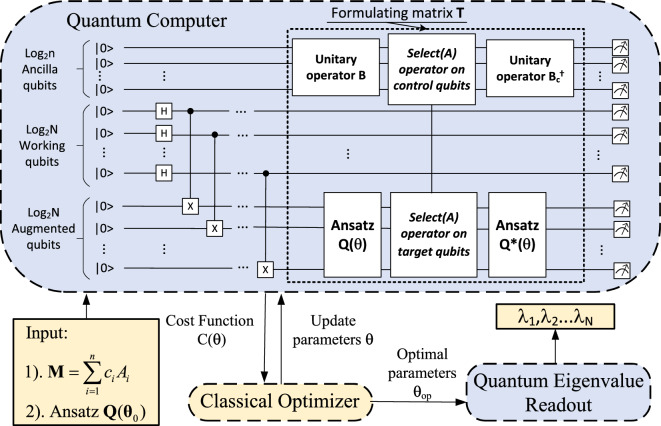
Schematic diagram of Variational quantum universal eigensolver (VQUE).

We devise the Variational Quantum Universal Eigensolver (VQUE) to empower the Variational Quantum Algorithms (VQA) to evaluate eigenvalues of non-Hermitian matrices. Our approach is underpinned by Schur’s decomposition theory, which assures the existence of a unitary matrix capable of triangularizing the non-Hermitian matrix. This unitary matrix is iteratively located using the VQA. To ensure the efficiency of this search, we introduce the Quantum Process Snapshot (QPS) method. Furthermore, we have developed a novel cost function to optimize matrix triangularization. This function is efficiently evaluated using the QPS method, and it not only enhances the original cost function in the Variational Quantum Eigensolver (VQE) method but also provides an additional measure of accuracy.

Figure 2 shows the schematic diagram of the introduced variational quantum universal eigensolver. To calculate the eigenvalue of an arbitrary matrix ${\varvec{M}}$, firstly, the classical computer prepares the inputs to the VQUE: (1) Decompose the matrix ${\varvec{M}}$ as a linear combination of unitaries ${\varvec{M}}=\sum _{i}^{}c_i{\varvec{A}}_i$; (2) Initialize the variational parameters $\varvec{\theta }_0$ for the ansatz circuit ${\varvec{Q}}(\varvec{\theta })$. After taking the inputs, VQUE enters an optimization process to train the ansatz ${\varvec{Q}}(\varvec{\theta })$ to triangularize the matrix ${\varvec{M}}$, i.e.,

17 $$ {\varvec{T}}={\varvec{Q}}(\varvec{\theta })^\dagger {\varvec{M}}{\varvec{Q}}(\varvec{\theta }) =\sum _{i}^{}c_i{\varvec{Q}}(\varvec{\theta })^\dagger {\varvec{A}}_i{\varvec{Q}}(\varvec{\theta }), $$

where matrix ${\varvec{A}}_i$ is unitary and thus can be directly realized by quantum gate operation.

By utilizing the QPS method above, the cost function $C(\varvec{\theta })$ in (18) is designed to quantify the triangulation extent:

18 $$\begin{aligned} C(\varvec{\theta })=\sum _{l=2}^{N}\sum _{m=1}^{l}(|\langle e_l|{\varvec{T}}|e_m\rangle |^2), \end{aligned}$$

where each $(|\langle e_l|{\varvec{T}}|e_m\rangle |^2)$ is just the possibility of measuring the quantum state $|e_{m\_l}\rangle$ (see the definition in (6)). Thus the summation is essentially counting the number of measurements $N_C$ which fall into the quantum states set ${\mathcal {C}}=\{|e_{m\_l}\rangle \big |\, \text{ where } \, l=2,3,\dots ,N \, \text{ and } \, m=1,2,\dots ,l\}$, compared with the total number of shots $N_{shots}$, i.e. $C(\varvec{\theta })\equiv N_{C}/N_{shots}$.

It is also crucial to take into account that ${\varvec{T}}$ is implemented probabilistically (see  (16)), thus the number of successful implementations $N_\mathrm{si}$ might be significantly less than the number of circuit executions $N_\mathrm{shots}$. However, insufficient $N_\mathrm{si}$ may create statistical fluctuation to evaluate $C(\theta )$, which makes it difficult to accurately compute its gradient. Fortunately, based on the theory proposed by Cerezo^22^, this challenge can be overcome by simply increasing a reasonable number of circuit executions, as the possibility of occurring large relative errors will **exponentially** reduced with a linearly increased $N_\mathrm{si}$. Therefore, in practice, we can measure all the qubits and repeat the experiment $N_\mathrm{shot}$ number of times to numerically check how large $N_\mathrm{shot}$ needs to be to achieve the required accuracy $\varepsilon$. In Section ‘Influence of Number of Circuit Executions’ of ’Parametric study’, this is demonstrated in the study of evaluating the eigenvalues of a 4 by 4 matrix.

From the measurement results by the quantum computer, the cost function $C(\varvec{\theta })$ can be computed. The parameters $\varvec{\theta }$ are then adjusted by an optimizer in the classical computer (e.g., gradient descent), which is used by the ansatz in the next round of the loop. It is easy to verify that when $C(\varvec{\theta })$ is approaching zero, so is (5), and thus the matrix ${\varvec{T}}$ can be quantified as close to a triangular matrix.

In theory, the target of VQUE is to find the parameters $\varvec{\theta }$ which minimizes the cost function $C(\varvec{\theta })$, In practice, however, the main interest is usually focused on finding a close enough solution, within a preset error threshold based on the application purpose. However, in the traditional VQE method, no quantification index is directly available, and the extent of optimization is unknown before finding the global optimum solution. This might become the main bottleneck for applying the traditional VQE technique to solve real-world problems, where the numerical error is expected to be within a specific range. Besides, even with extensive effort, phenomena like the barren plateau^36^ and noise introduced in NISQ devices might still deeply influence the optimization performance, thus the optimized solution might be impossible to find. Efforts are still being paid to mitigate the barren plateau issues^37^.

This challenge is partially overcome by the cost function introduced in (18), whose value is designed as a quantification index of a triangular matrix. The more a matrix is triangularized, the closer its diagonal entries are to its eigenvalues. Thus a customized error threshold $\varepsilon$ can be used as a termination condition for the optimization process, as follows

19 $$\begin{aligned} C(\varvec{\theta }_{op})\le \varepsilon . \end{aligned}$$

Once the termination condition is reached with ${\varvec{Q(\theta _{op})}}$, the diagonal elements of the matrix ${\varvec{T}}={\varvec{Q(\theta _{op})}}^\dagger {\varvec{MQ(\theta _{op})}}$ are used as the estimate of the eigenvalues of the original matrix. Therefore, performing the Hadamard test to each unitary ${\varvec{Q(\theta _{op})}}^\dagger {\varvec{A_iQ(\theta _{op})}}$ (note ${\varvec{M}}=\sum _{i}^{}c_i{\varvec{A}}_i$) with the intial state $|e_i\rangle$, the real and imagery parts of $i^{th}$ eigenvalue can be evaluated.

It is also worth noting that the algorithm designed here is to reach out to all eigenvalues similar to the classical eigenvalue algorithm. It might be possible to adapt and modify this method if a specific subset of eigenvalues are the main interest.

### Resource analysis

In this subsection, we analyze the escalation of computational resources in correlation with the expansion of the network size. Consider an *N*-dimensional matrix that can be decomposed into the sum of *n* unitaries. Quantum hardware possesses the ability to store an *N*-dimensional vector and perform matrix operations using only $O(log_{2}N)$ qubits, requiring exponentially fewer resources compared to classical computers. The computational complexity of executing VQUE in each iteration to triangularize the matrix approximates to $O(log_{2}N+n^{2})$. This complexity encompasses: 1). $O(log_{2}N)$ gates for the execution of the quantum process snapshot 2). $O(n^{2})$ gates for the implementation of the non-unitary gate operation 3). Additional gates required for the ansatz. Consequently, if a matrix can be represented by a limited number of unitaries (i.e., $N>>n$), the proposed VQUE could potentially offer an exponential quantum advantage in matrix triangularization. However, the quantum advantage will diminish to linear to produce all *N* eigenvalues. It is critical to underscore that, akin to all other Variational Quantum Algorithms (VQAs), the extent of quantum advantage conferred by VQUE is largely contingent on the optimization process.

**Figure 3:**
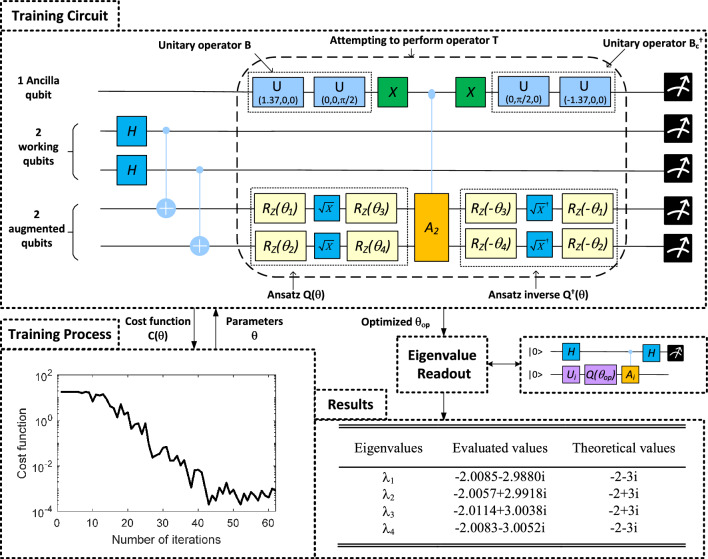
Example 1a: Simple illustrative example in noise-free quantum simulator.

## Parametric study

In this section, we showcase the numerical results from our VQUE implementation. First, to validate the devised method, we implement the VQUE on IBM’s noise-free quantum simulator to assess the eigenvalues of a non-Hermitian matrix. Following this validation, we deploy the VQUE on a real IBM quantum computer (ibmq-quito, 5 qubits, 16 quantum volume). To evaluate scalability and universality, we apply the VQUE to a 1024-dimensional matrix and a non-diagonalizable matrix. To primarily emphasize the performance assessment of the algorithm, we conduct these tests on the noise-free quantum simulator. Ultimately, we employ our algorithm for the small signal analysis of an actual power network.

### Verification of VQUE on by a simple illustrative example

We will demonstrate our method with a simple example via a quantum simulator and using a real quantum device.

#### Implementation in noise-free quantum simulator

The goal is to minimze the cost function in (18) and obtain the optimal parameters,

20 $$\begin{aligned} \varvec{\theta }_{op}=arg\min _{\varvec{\theta }}C(\varvec{\theta }). \end{aligned}$$

In this subsection, we use the VQUE approach to evaluate eigenvalues of the matrix ${\varvec{M}}$ defined as follows,

$$\begin{aligned} {\varvec{M}}= \begin{bmatrix} -2 &{} 0 &{} 0 &{} -3\\ 0 &{} -2 &{} 3 &{} 0\\ 0 &{} -3 &{} -2 &{} 0\\ 3 &{} 0 &{} 0 &{} -2\\ \end{bmatrix}, \end{aligned}$$

Applying (2) decomposes matrix ${\varvec{M}}$ as a linear combination of unitaries ${\varvec{M}}=-2\cdot ({\varvec{\sigma _{I}\otimes \sigma _{I}}})+3\cdot ({\varvec{\sigma _{y}\otimes \sigma _{x}}})$. Note there is a negative coefficient -2 in this decomposition, thus the operator ${\varvec{M}}$ could not be directly implemented by the technique presented in^34^. To implement the matrix in a quantum circuit, firstly, we perform the square root operation on the coefficient vector $$\begin{pmatrix} -2 \\ 3 \end{pmatrix}$$, giving $$\begin{pmatrix} 1.4142j \\ -1.7321 \end{pmatrix}$$, which is then normalized to $$\begin{pmatrix} 0.6325j \\ -0.7746 \end{pmatrix}$$ by a constant 2.2361.

To search the unitary matrix ${\varvec{Q}}$ which triangularizes the matrix (i.e. ${\varvec{T}}={\varvec{Q}}{\varvec{M}}{\varvec{Q^{\dagger }}}$), 5 qubits are used as shown in Fig. 3. We use the RZ-SX-RZ gates to form the variational circuit, which results in an ansatz with 4 parameters.

Firstly, the ${\varvec{B}}$ circuit defined by (11) converts the ancilla qubit to the quantum state:

21 $$\begin{aligned} |\psi \rangle _a=0.6324j|0\rangle -0.7746|1\rangle , \end{aligned}$$

In the meanwhile, the quantum augmentation circuit efficiently prepares a quantum state:

22 $$ \begin{aligned} |\Psi \rangle _{w \& a}=\frac{1}{2}(|0000\rangle +|0101\rangle +|1010\rangle +|1111\rangle ), \end{aligned}$$

which corresponds to the vectors: $$\begin{pmatrix}\vec {e_1} \\ \vec {e_2} \\ \vec {e_3}\\ \vec {e_4} \end{pmatrix} $$ and $\vec {e_i}$ is a 4-dimensional unit vector with 1 in its i-th entry.

The ansatz ${\varvec{Q(\theta )}}$ is then performed to implement the matrix transformation ${\varvec{T}}={\varvec{Q(\theta )^{\dagger }}}{\varvec{M}}{\varvec{Q(\theta )}}$. Subsequently, we perform the matrix formulation circuit that kickbacks the phase term in (21) to the corresponding operators $({\varvec{\sigma _{I}\otimes \sigma _{I}}})$ and $({\varvec{\sigma _{y}\otimes \sigma _{x}}})$, respectively. In the end, the inverse ansatz circuit (${\varvec{Q(\theta )^{\dagger }}}$) and the inverse circuit of ${\varvec{B_{c}}}$ (i.e., ${\varvec{B_c}}^{\dagger }$) are applied to the augmented qubits and ancilla qubits, respectively, which yields the quantum state:

23 $$ \begin{aligned} \begin{aligned} \frac{1}{5}|0\rangle _{ancilla}\otimes {\varvec{T}}|\Psi \rangle _{w \& a}+\sqrt{1-\frac{1}{5^2}}|\phi \rangle , \end{aligned} \end{aligned}$$

where the ancillary state $|\phi \rangle$ is supported in the subspace orthogonal to $|0\rangle$ and 5 is the coefficient *s* calculated from (16).

Therefore, if the ancilla qubit is measured in state $|0\rangle$, operator ${\varvec{T}}$ is successfully applied to working and augmented qubits, which corresponds to the vector $$\begin{pmatrix} {\varvec{T}}\vec {e_1} \\ {\varvec{T}}\vec {e_2} \\ {\varvec{T}}\vec {e_3}\\ {\varvec{T}}\vec {e_4} \end{pmatrix}$$. Subsequently, by counting the frequency of measuring $|0001\rangle ,|0010\rangle ,|0011\rangle , |0110\rangle ,|0111\rangle ,|1011\rangle$ in the working and augmented qubits, the cost function $C(\varvec{\theta })$ defined in (18) is evaluated. For instance, the quantum state $|0001\rangle$ is related to the vector $$\begin{pmatrix} \vec {e_2} \\ \vec {0} \\ \vec {0}\\ \vec {0} \end{pmatrix}$$, thus the frequency of getting this result corresponds to the magnitude of entry $T_{21}$, i.e.:

24 $$ \begin{aligned} \begin{aligned} |T_{21}|^2=\Big |\begin{pmatrix}\vec {e_2}&\vec {0}&\vec {0}&\vec {0}\end{pmatrix}\begin{pmatrix}{\varvec{T}}\vec {e_1} \\ {\varvec{T}}\vec {e_2} \\ {\varvec{T}}\vec {e_3}\\ {\varvec{T}}\vec {e_4}\end{pmatrix}\Big |^2\\ \equiv |\langle 0001|{\varvec{T}}|\Psi \rangle _{w \& a}|^2\cdot c, \end{aligned} \end{aligned}$$

where $$c=s\cdot s \cdot (2)^2=100$$.

After the cost function is evaluated, “Constrained Optimization By Linear Approximation optimizer”^38^ is used to update the parameters ${\varvec{\theta }}$, which will then be used in the ansatz ${\varvec{Q(\theta )}}$ in the next iteration. Figure 3 plots the cost function versus the number of iterations. It shows that the cost function is consistent below 0.01 after around 140 iterations. Thus the ${\varvec{\theta }}$ after 155 iteration is selected as ${\varvec{\theta _{op}}}$ .

The Hadmard test is then applied with the optimized ansatz ${\varvec{Q(\theta _{op})}}$ to evaluate the $i^{th}$ eigenvalue. The evaluated eigenvalues are shown in the results part in Fig. 3. Compared with the theoretical eigenvalues, $\lambda _3$ has the largest relative error of $0.39\%$, which is quite close to the theoretical one. Therefore, we conclude that:

- In theory, the devised method has the capability to evaluate the eigenvalues of a general matrix with complex eigenvalues.
- As the absolute error is similar for all eigenvalues, the evaluation of smaller eigenvalues might have larger relative errors.

#### Influence of number of circuit executions

As discussed in section ‘Variational quantum universal eigensolver’ of ‘results’, the number of circuit executions $N_\mathrm{shots}$ plays a key role in accurately evaluating the eigenvalues. As pointed out by Cerezo^22^, the possibility of resulting large evaluation errors should exponentially decrease with a linear increased $N_\mathrm{shots}$. To confirm this theoretical analysis, we make a numerical study in the same quantum circuit as shown in Fig. 3.

**Figure 4:**
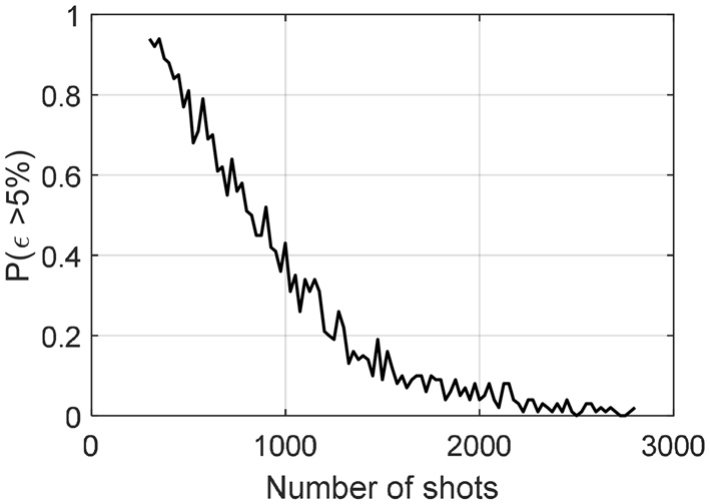
VQUE accuracy study.

In the experiment, we set the accuracy threshold as the maximum error in the eigenvalue evaluations is less than $5\%$. From the experiment, the algorithm starts to make meaningful estimation with $N_\mathrm{shots}=300$. Thus we increased the number of shots $N_\mathrm{shots}$ from 300 to 2800, with a step of 25. For each $N_\mathrm{shots}$, we repeat 100 times and count the number of results which gives the eigenvalues inside the accuracy threshold. The experiment result is shown in Fig. 4.

It can be observed that with the linearly increased number of $N_\mathrm{shots}$, the possibility for VQUE to make estimations with maximum relative error larger than $5\%$ is close to exponentially decreased. Therefore, it is reasonable to expect overcoming the challenge of ${\varvec{T}}$ is implemented probabilistically by an acceptable increase of $N_\mathrm{shots}$.

#### Implementation on real quantum machine

**Figure 5:**
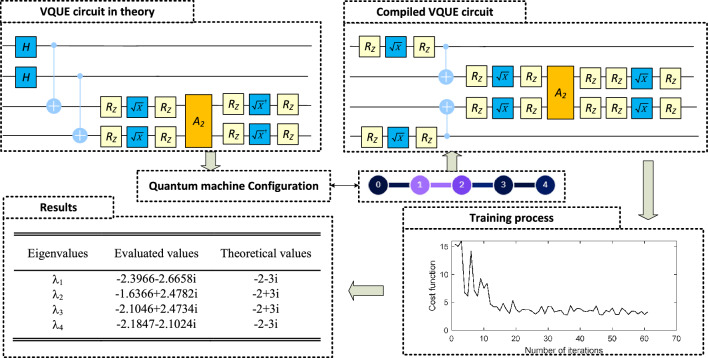
Example 1b: Illustrative example in real quantum machine.

In this subsection, we implement example 1 in a real quantum machine to study its performance in the current Noisy Intermediate-Scale Quantum (NISQ) environment. We use the IBM quantum computer ibmq_manila, which is a 32-quantum volume machine with 5 qubits. Its structure is shown in the quantum machine configuration part in Fig. 5.

As shown in Fig. 5, due to the limited connectivity of the real quantum computer, the quantum entanglement between the ancilla qubit and augmented qubits will require a significant number of CNOT gates to realize in the quantum computer. Therefore, we made an engineering trick here by removing the ancilla qubit and only performing ${\varvec{\sigma _{y}\otimes \sigma _{x}}}$ to the augmented qubits as shown in the VQUE circuit in theory in Fig. 5. Such treatment is based on the fact that ${\varvec{Q}}^\dagger {\varvec{I}}{\varvec{Q}}={\varvec{I}}$ for an arbitrary unitary matrix ${\varvec{Q}}$. Therefore, searching for the ansatz to triangularize the matrix ${\varvec{M}}=-2\cdot ({\varvec{\sigma _{I}\otimes \sigma _{I}}})+3\cdot ({\varvec{\sigma _{y}\otimes \sigma _{x}}})$ is equivalent to finding the ansatz which triangularize the matrix ${\varvec{\sigma _{y}\otimes \sigma _{x}}}$.

The circuit is then compiled to fit the configuration of the quantum machine as shown in Fig. 5. It can be observed that the noise introduced by the quantum computer will significantly influence the classical optimizer. As a result, the cost function can only converge to a local minimum of around 2, which results in the estimated eigenvalues having a relative error from $14.38\%$ to $25.42\%$.

### VQUE scalability demontration

**Figure 6:**
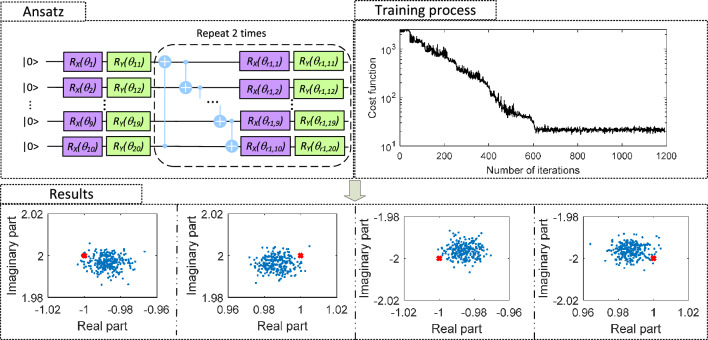
Example 2: VQUE scalability demonstration.

In this subsection, the VQUE algorithm estimates the eigenvalues of a 1024-dimensional matrix in the noise-free quantum simulator. The matrix is composed of two unitary matrices: ${\varvec{\sigma _{I}\otimes \sigma _{I}\otimes \sigma _{I}\otimes \sigma _{I}\otimes \sigma _{I}\otimes \sigma _{I}\otimes \sigma _{I}\otimes \sigma _{I}\otimes \sigma _{I}\otimes \sigma _{z}}}$ and ${\varvec{\sigma _{I}\otimes \sigma _{I}\otimes \sigma _{z}\otimes \sigma _{I}\otimes \sigma _{x}\otimes \sigma _{I}\otimes \sigma _{I}\otimes \sigma _{z}\otimes \sigma _{I}\otimes \sigma _{I}}}$, with coefficients $-1$ and 2 respectively. An entangled RX-RY gates ansatz as shown in Fig. 6 is selected as the parameterized circuit. As shown in the training process, the cost function is converged to 20, which implies the average value below the diagonal elements is around $3.38184\cdot 10^{-5}$. It has 1024 eigenvalues in the values set of $\{-1+2j,-1-2j,1+2j,1-2j\}$. The estimated eigenvalues are shown in the results part of Fig. 6. It can be observed that all the estimated eigenvalues are within the relative error of $2\%$, which maintains a very high estimation accuracy. This indicates that VQUE has outstanding scalability and thus brings a huge potential to resolving ultra-large size eigenvalue problems.

### Verification of VQUE for non-diagonalizable matrix

In this subsection, to demonstrate the universality, the VQUE is applied to evaluate the eigenvalue of a non-diagonalizable matrix ${\varvec{N}}$:

$$\begin{aligned} {\varvec{N}}= \begin{bmatrix} 5 &{} 4 &{} 2 &{} 1\\ 0 &{} 1 &{} -1 &{} -1\\ -1 &{} -1 &{} 3 &{} 0\\ 1 &{} 1 &{} -1 &{} 2\\ \end{bmatrix}, \end{aligned}$$

**Figure 7:**
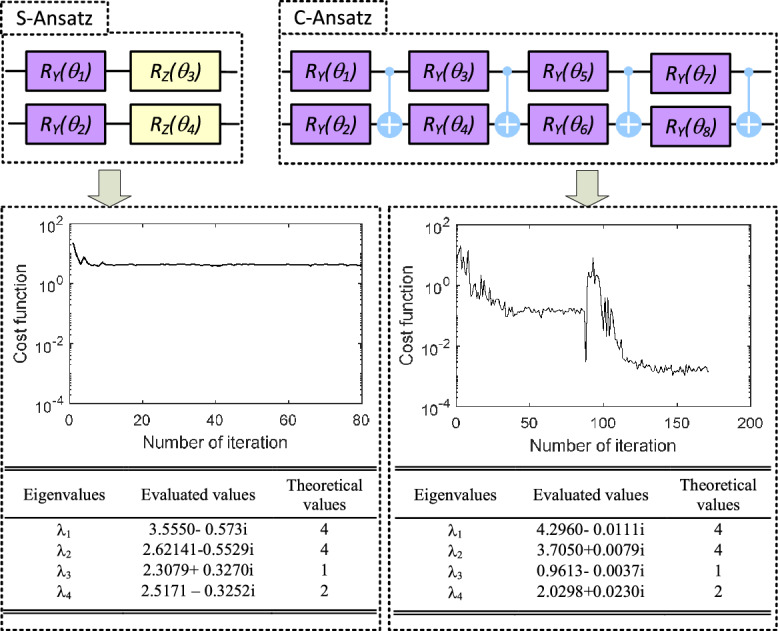
Example 3: Eigenvalue evaluation of non-diagonalizable matrix.

It is easy to verify that this matrix only has three linear independent eigenvectors and thus is not similar to any diagonal matrices^5^. Decomposing the matrix by (2) gives the 13 coefficients, which is significantly more complicated than the matrix used in the previous sections. As shown in Fig. 7, when the same ansatz as in previous cases is used, the cost function could only converge around 4, which is too far away from a triangular matrix. By this ansatz, the evaluated eigenvalues are shown on the left side of the results in Fig. 7. It can be observed that $\lambda _3$ has the largest evaluation error of $\epsilon =1.3079-0.3270$, which is $134.82\%$ of its original eigenvalue. This indicates that the original eigenvalue $\lambda _3$ is submerged by the error, which is consistent with the conclusion we have drawn from the cost function.

To precisely evaluate the eigenvalues, we slightly modify the ansatz as shown in the “C-Ansatz” of Fig. 7. This complicated ansatz enables the VQUE to search for the solution in a much larger subspace. With the empowered ansatz, the cost function could then be brought down to 0.001 after 150 iterations. By this optimized ansatz, the eigenvalues are listed on the right side of the results in Fig 7. At this time, the largest relative error appears in eigenalue $\lambda _1$, which is $7.37\%$ of original eigenvalue. Compared the results with the one from the simple anstanz in the beginning, the eigenvalue evaluation accuracy is dramatically improved from $134.82\%$ error to $7.37\%$ error.

It is essential to realize that it is our devised method that enables such a trial-and-error process. In the original version of VQE, only the smallest eigenvalue is available, and there is no indication of how close the evaluated eigenvalue is to the theoretical one. This is critical in practical applications, where the numerical error is expected to be controlled inside a specific range. However, in today’s NISQ machines, the variational method is not necessarily guaranteed to work. For instance, the barren plateau phenomenon will require exponential precision to train the ansatz. Besides, a universal parameterized circuit itself requires a very complex structure with a tremendousnumer of parameters (e.g.^39^ introduced a depth 7, 24 parameters universal ansatz for 2 qubits). Such complexity will not only be deeply influenced by the implementation noise but also increase the possibility of converging to a sub-optimum during non-convex optimization^23^.

In the standard VQE approach, regardless of the ansatz chosen, the algorithm consistently yields an eigenvalue estimate. However, it does not provide insights into the accuracy of the predicted eigenvalue, necessitating the use of a sufficiently comprehensive ansatz to guarantee a dependable evaluation. In contrast, the cost function within our proposed methodology serves a dual purpose, also acting as an indicator of accuracy. As shown in example 3, this feature allows for the exploration of a shallower tentative ansatz through a process of trial and error, as it offers immediate feedback on the reliability of the results. Our introduced method thus opens an engineering window to solve the problem without explicitly overcoming all the challenges above. A threshold value can be set based on the specific application. We could then start with a simple ansatz and gradually increase its complexity to attempt to reduce the $C(\varvec{\theta })$ below the threshold value. If a solution that matches the requirement could be found in the subspace spanned by the ansatz, there is no need to search the whole space for a theoretical solution. This process can be further improved and automated by the recent developed reinforcement learning-based methods for quantum architecture search^40^.

### Verification of VQUE for power network stability and oscillation mode analysis

**Figure 8:**
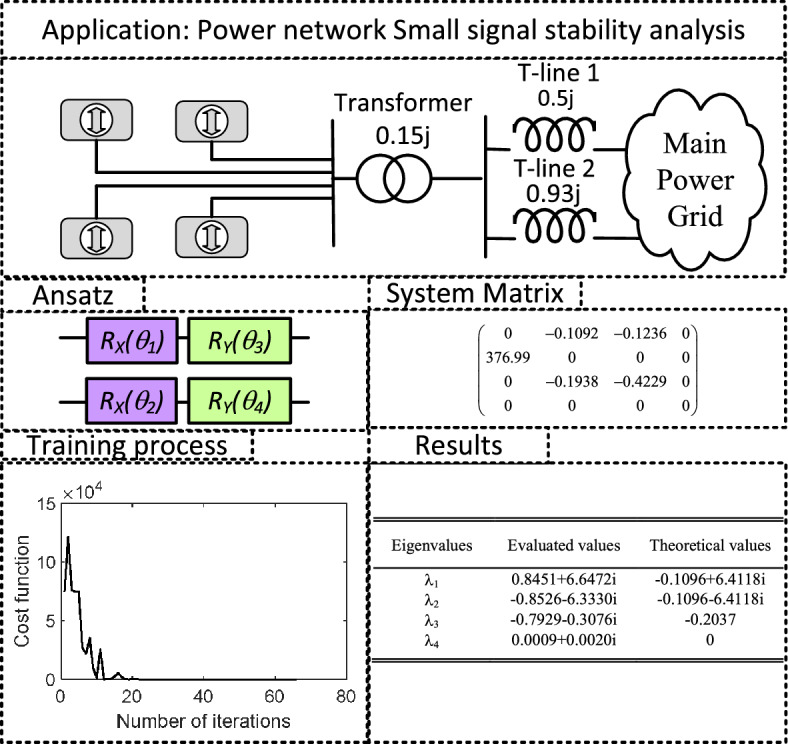
Example 4: Verification of VQUE for power network stability and oscillation mode analysis.

In this subsection, VQUE is used to analyze a real-world small signal stability problem of power network^1^. As shown in Fig. 8, a thermal generating station consisting of four generators is connected to the main power grid through the transformer and two transmission lines: T-line 1 and T-line 2. A more detailed introduction of system parameters can be found in^1^ on pages 753 and 754. To analyze the small-signal stability characteristics of the system when the T-line 2 is tripped, the system matrix ${\varvec{M}}$ is formed as shown in Fig. 8. Decomposing the matrix by (2) gives 16 unitary matrices, with $s=377.730318$ in (16). As shown in the training process in Fig. 8, a simple ansatz could easily bring the cost function exactly to zero after 40 interactions, with 1 million shots. However, from the eigenvalue evaluation results, it can be seen that only the large imaginary parts in eigenvalues $\lambda _1$ and $\lambda _2$ are precisely predicted, which is directly related to the system oscillation modes.

Note the system is considered to be small signal stable, if and only if all of its eigenvalues are on the left-hand side. However, the real part of $\lambda _1$ is small and is distorted to be positive by VQSU, the system stability conclusion might be misleading.

## Challenges for future work

In this paper, we opened the door for the variational quantum algorithm to tackle the eigenvalue estimation of a general matrix. Meanwhile, numerical improvement is still required to make this algorithm shinning in practical applications. To pave the path for a future robust VQUE algorithm, in this section, we list the main challenges that are obstacles to the algorithm for scaling.

### Excessive number of shots requirement

In the system matrix such as shown in Fig. 8, the smallest magnitude entry is -0.1092, which is only $0.1\%$ of its largest entry. However, if we ignore this entry, the eigenvalue will become $\lambda _1=1.9486, \lambda _2=-1.1892 + 1.7945j, \lambda _3=-1.1892 - 1.7945i, \lambda _4=0$, which is almost completely different with the original eigenvalues. Therefore, extremely high precision is needed in the training process. However, based on Hoeffding’s inequality^22^, this implies an increasing number of shots. Fortunately, as shown in section ’Influence of number of circuit executions’ of ’Parametric study’, such a requirement is usually easy to accommodate, as the possibility of resulting large relative error will exponentially be reduced with an increasing number of shots.

Besides, when the matrix is decomposed as a linear combination of unitary matrices, the summation of its coefficients magnitude *s* might also be very large. As shown in (16), this will quadratically decrease the possibility of implementing the non-unitary operator. Modifications like cooperating with the idea of Amplitude amplification^41^ to boost the success rate can be implemented to improve this issue.

### Unitary decomposition challenge

The system information is usually embedded in the network matrix for many practical applications. To enable evaluating its eigenvalues in a quantum computer, it needs to be decomposed as a linear combination of unitary matrices. Although (2) provides a systematical way to conduct this decomposition, its computational cost for the decomposition itself is very high and usually results in a $O(N^2)$ number of coefficients. In this case, even the classical VQE needs to implement $O(N^2)$ times, which eliminates the quantum advantage. Therefore, it is critical to develop an efficient way to implement the unitary decomposition with a minimum number of unitary matrices.

It is also valuable to note that this issue exists for many variational quantum algorithms used for scientific computations, where the targeted matrix is not naturally formed as a summation of directly realizable unitary operators. As pointed out by Aaronson^42^, the matrix implementation issue is also considered as one of the main caveats for HHL algorithm, which is crucial for practical applications.

### Optimization challenge

The introduced quantum eigenvalue algorithm belongs to the class of hybrid quantum-classical algorithms. It involves the minimizing cost function procedure, which depends on the optimizer in the classical computer. However, the resulting optimization problem is in general nonconvex, it is common that VQUE converges to suboptimal parameter values. Besides, if the algorithm is implemented on a real quantum machine as in section Implementation on real quantum machine, the noise will also significantly influence its convergence. To temporarily overcome this issue on a noise-free quantum simulator, we repeat the optimization procedure many times with different initial parameters. Novel techniques to improve the optimization process (e.g.^23^) as well as mitigate errors are critical to enabling the method robustly tackle real-world challenges.

### State preparation

This algorithm involves the state-preparing process when implementing the non-unitary operators. It is valuable to notice that, the dimension of the quantum state is directly related to the number of unitary matrices (i.e., not the dimension of the matrix). In this paper, we use the embedded function in IBM qiskit^43^ for preparing the state, which is currently still computationally expensive^42^. This area is still an ongoing research topic, and many efficient algorithms (e.g. QRAM^44^) are expected to be available in the short future.

## Discussion

This paper introduced the variational quantum universal eigensolver (VQUE) algorithm, which enables the quantum computer to estimate the eigenvalues of a general matrix. VQUE method unitarizes the eigenvalue problem by Schur decomposition theory and maintains the quantum advantage by the introduced quantum process snapshot (QPS) technique. We also devised a novel way to directly implement a non-unitary matrix in the QPS quantum circuit, which empowers VQUE to estimate the eigenvalues of a general non-unitary matrix. By an illustrative example, VQUE is validated on the noise-free simulator and a real quantum machine. Its scalability and universality are tested through a 1024-dimensional matrix and a non-diagonalizable matrix in the noise-free simulator, respectively. We also reveal its numerical challenges through a real-world application example. This is the first realizable quantum algorithm in today’s NISQ devices to evaluate the eigenvalues of a general matrix. It shows outstanding scalability and opens a window to enable quantum computers directly tackle real-world ultra-scale problems, thus moving the quantum algorithm frontier one step closer to practical applications.

## Methods

### Proof of non-unitary gate operation implementation

In section ’Non-unitary gate operation’ of ’Results’, we devised the non-unitary operation based on the idea introduced in^34^. The mathematical proof of such implementation in (16) is provided below:

#### Proof

From (13), we have:

25 $$\begin{aligned} \begin{aligned} {\varvec{W_m}}(|0\rangle \otimes |\psi \rangle )={\varvec{B_c}}^\dagger \otimes {\varvec{I}}\Big (\frac{1}{\sqrt{s}}\sum _{i=0}^{2^n-1}\sqrt{c_i}|i\rangle \otimes {\varvec{A}}_i|\psi \rangle \Big ). \end{aligned} \end{aligned}$$

As defined in (15) (i.e., ${\varvec{B_c}}|0\rangle =\frac{1}{\sqrt{s}}\sum _{i=0}^{2^n-1}(\sqrt{c_i})^*|i\rangle$), the operator ${\varvec{B_c}}$ can be represented as:

26 $$\begin{aligned} \begin{aligned} {\varvec{B_c}}=\frac{1}{\sqrt{s}}\sum _{i=0}^{2^n-1}(\sqrt{c_i})^*|i\rangle \langle 0|+{\varvec{B_R}} \end{aligned} \end{aligned}$$

where the superscript * denotes complex conjugate and the magnitude of vector ${\varvec{B_R}}|0\rangle$ is zero. Therefore, its complex conjugate transpose ${\varvec{B_c}}^\dagger$ is:

27 $$\begin{aligned} \begin{aligned} {\varvec{B_c}}^\dagger =\frac{1}{\sqrt{s}}\sum _{i=0}^{2^n-1}\sqrt{c_i}|0\rangle \langle i|+{\varvec{B_R}}^\dagger \end{aligned} \end{aligned}$$

Substituting (27) to (25) gives:

28 $$\begin{aligned} \begin{aligned} {\varvec{W_m}}(|0\rangle \otimes |\psi \rangle )=\Big ( \frac{1}{s}\sum _{i=0}^{2^n-1}c_i|i\rangle \otimes {\varvec{A}}_i|\psi \rangle \Big )+\Big ({\varvec{B_R}}^\dagger \frac{1}{\sqrt{s}}\sum _{i=0}^{2^n-1}\sqrt{c_i}|i\rangle \otimes {\varvec{A}}_i|\psi \rangle \Big ) \equiv \frac{1}{s}|0\rangle \otimes {\varvec{M}}|\psi \rangle +\sqrt{1-\frac{1}{s^2}}|\phi \rangle \end{aligned} \end{aligned}$$

where $\sqrt{1-\frac{1}{s^2}}|\phi \rangle \equiv {\varvec{B_R}}^\dagger (\frac{1}{\sqrt{s}}\sum _{i=0}^{2^n-1}\sqrt{c_i}|i\rangle \otimes {\varvec{A}}_i|\psi \rangle$ and the ancillary state in state $|\phi \rangle$is supported in the subspace orthogonal to $|0\rangle$ as $\langle 0|{\varvec{B_R}}^\dagger |i\rangle \equiv 0,\forall {i}\in {0,1...2^n-1}$, which results from the definition in (26) that the magnitude of vector ${\varvec{B_R}}|0\rangle$ is zero.

Q.E.D. $$\square $$

## Data Availability

The simulation datasets used during the current study are available from the corresponding author upon reasonable request.
